# Cabins, castles, and constant hearts: rhythm control therapy in patients with atrial fibrillation

**DOI:** 10.1093/eurheartj/ehz782

**Published:** 2019-11-22

**Authors:** Stephan Willems, Christian Meyer, Joseph de Bono, Axel Brandes, Lars Eckardt, Arif Elvan, Isabelle van Gelder, Andreas Goette, Michele Gulizia, Laurent Haegeli, Hein Heidbuchel, Karl Georg Haeusler, Josef Kautzner, Lluis Mont, G Andre Ng, Lukasz Szumowski, Sakis Themistoclakis, Karl Wegscheider, Paulus Kirchhof

**Affiliations:** 1 AFNET, Münster, Germany; 2 Department of Cardiology, Asklepios Kliniken St Georg, Hamburg, Germany; 3 Semmelweis University, Budapest, Hungary; 4 Department of Cardiology, Electrophysiology, University Heart Center, University Hospital Hamburg-Eppendorf, Hamburg, Germany; 5 DZHK (German Centre for Cardiovascular Research), partner site Hamburg/Kiel/Lübeck, Hamburg, Germany; 6 Institute of Cardiovascular Sciences, University of Birmingham and University Hospitals Birmingham NHS Foundation Trust, IBR 136, Wolfson Drive, Birmingham, UK; 7 Department of Clinical Research, University of Southern Denmark, and Odense University Hospital, Odense, Denmark; 8 Department of Cardiology II—Electrophysiology, University Hospital Münster, Münster, Germany; 9 Isala Diagram B.V. SMO Zwolle, Zwolle, Netherlands; 10 University of Groningen, University Medical Center Groningen, Groningen, Netherlands; 11 St. Vincenz Hospital Paderborn, Cardiology and Intensive Care Medicine, Paderborn, Germany; 12 Garibaldi-Nesima Hospital, Catania, Italy; 13 Universitätsspital Zürich, Zürich, Switzerland; 14 Medical University Department, Kantonsspital Aarau, Aarau, Switzerland; 15 University Hospital Antwerp, Antwerp, Belgium; 16 Department of Neurology, University Hospital Würzburg, Würzburg, Germany; 17 Institute for Clinical and Experimental Medicine (IKEM), Prague, Czech Republic; 18 Hospital Clinic Barcelona, Barcelona, Spain; 19 National Institute for Health Research Leicester Biomedical Research Centre, University of Leicester, Glenfield General Hospital, Leicester, UK; 20 National Institute of Cardiology, Warsaw, Poland; 21 Unit of Electrophysiology and Cardiac Pacing, Ospedale Dell'Angelo, Venice, Italy; 22 Department of Medical Biometry and Epidemiology, University Medical Center Hamburg-Eppendorf, Hamburg, Germany; 23 Sandwell and West Birmingham NHS Trust, Birmingham, UK

**Keywords:** Atrial fibrillation, Rhythm control therapy, AF ablation, Antiarrhythmic drugs, Heart failure, Stroke, Mortality

## Abstract

Recent innovations have the potential to improve rhythm control therapy in patients with atrial fibrillation (AF). Controlled trials provide new evidence on the effectiveness and safety of rhythm control therapy, particularly in patients with AF and heart failure. This review summarizes evidence supporting the use of rhythm control therapy in patients with AF for different outcomes, discusses implications for indications, and highlights remaining clinical gaps in evidence. Rhythm control therapy improves symptoms and quality of life in patients with symptomatic AF and can be safely delivered in elderly patients with comorbidities (mean age 70 years, 3–7% complications at 1 year). Atrial fibrillation ablation maintains sinus rhythm more effectively than antiarrhythmic drug therapy, but recurrent AF remains common, highlighting the need for better patient selection (precision medicine). Antiarrhythmic drugs remain effective after AF ablation, underpinning the synergistic mechanisms of action of AF ablation and antiarrhythmic drugs. Atrial fibrillation ablation appears to improve left ventricular function in a subset of patients with AF and heart failure. Data on the prognostic effect of rhythm control therapy are heterogeneous without a clear signal for either benefit or harm. Rhythm control therapy has acceptable safety and improves quality of life in patients with symptomatic AF, including in elderly populations with stroke risk factors. There is a clinical need to better stratify patients for rhythm control therapy. Further studies are needed to determine whether rhythm control therapy, and particularly AF ablation, improves left ventricular function and reduces AF-related complications.

**Figure:**
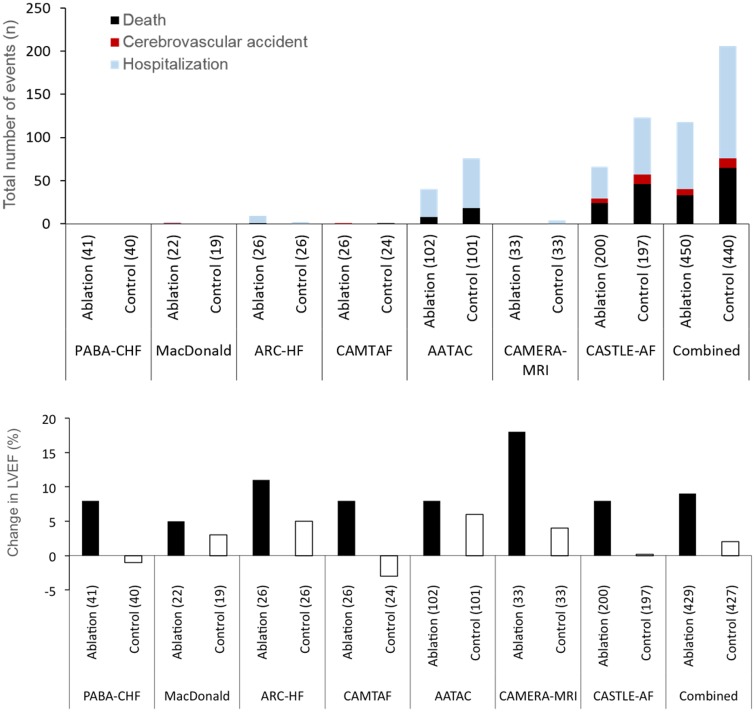

## Introduction

The prevalence of atrial fibrillation (AF) and its associated mortality and morbidity are expected to double or triple within the next two to three decades, driven by population ageing and increased incidence of AF.^1^^,^^2^ Even on optimal anticoagulation and rate control therapy, patients with AF are at high risk of cardiovascular death, particularly sudden death and death due to heart failure.^3^^,^^4^ Rhythm control therapy using antiarrhythmic drugs, cardioversion, and AF ablation, is clinically used to improve AF-related symptoms.^5^ Currently, there is no established indication for rhythm control therapy apart from improvement of AF-related symptoms.^6–8^ The CABANA (Catheter Ablation vs. Anti-arrhythmic Drug Therapy for Atrial Fibrillation) trial recently provided new confirmation on the safety of AF ablation in contemporary AF patients at risk of stroke.^9^ The smaller CASTLE-AF (Catheter Ablation vs. Standard Conventional Therapy in Patients with Left Ventricular Dysfunction and Atrial Fibrillation) suggests that AF ablation could improve outcomes in patients with AF and severe heart failure compared to drug therapy, combining rate control therapy and antiarrhythmic drug therapy.^10^ Here, we review the available evidence supporting the use of rhythm control therapy in patients with AF, discuss potential implications for indications, and highlight clinical evidence gaps.

## Rhythm control therapy improves atrial fibrillation-related symptoms

Restoring and maintaining sinus rhythm indicated to minimize symptoms is a main goal in patients who remain symptomatic despite adequate rate control.^11^^,^^12^ Interestingly, the effects of rhythm control on quality of life are less uniform than their clear effects on maintaining sinus rhythm (*Table 1*). Both natural variation in patient-reported quality of life, imprecise instruments to assess quality of life, and variable effects of rhythm control therapy on quality of life in individual patients can explain this heterogeneity.^13^^,^^14^ The European Heart Rhythm Association (EHRA) symptom score was introduced in 2007 as a simple clinical tool to quantify AF-related symptoms,^15^ with subsequent refinement and validation.^16^ Several disease-specific instruments are available, all with specific strengths and limitations.^17^ In addition, perceived AF-related symptoms may not always be due to AF, and concomitant cardiovascular diseases and risk factors may affect patient’s health perception in addition to the arrhythmia itself.^18^^,^^19^ Furthermore, patients with paroxysmal AF can be expected to report variable quality of life depending on their rhythm at the time of assessment, on their ability to memorize past symptoms during clusters of AF episodes, and by anxiety related to future episodes of AF.

**Table 1:** Effects of rhythm control therapy using antiarrhythmic drugs in controlled clinical trials

	PIAF	CTAF	RACE	AFFIRM	STAF	SAFE-T	AF-CHF	ATHENA	Flec-SL
Year of publication	2000	2000	2002	2002	2003	2005	2008	2009	2012
Number of patients	252	403	522	4060	200	665	1376	4628	635
Mean age	60	65	68	70	66	67	67	72	64
Sex	73% male	56% male	64% male	61% male	64% male	99% male	81% male	53% male	66% male
Inclusion criteria	Symptomatic persistent AF <1 year duration	Symptomatic AF eligible for antiarrhythmic drug therapy	Recurrent persistent AF <1 year duration	>65 years or <65 years with additional risk factor for stroke with AF likely to be recurrent and likely to cause illness or death	Persistent AF either >4 weeks or enlarged LA or heart failure	Persistent AF on anticoagulation	Symptomatic HF (NYHA II–IV), LVEF <36%	Patients with AF, and >70 years with one comorbidity or >75 years	Patients undergoing planned cardioversion
Exclusion criteria	NYHA IV, unstable angina	NYHA III–IV, severe CKD, QTc >0.48	NYHA IV, previous amiodarone, pacemaker	Reversible cause of AF	Permanent AF >2 years, paroxysmal AF	NYHA III–IV, CKD, initially AF >12 months (eliminated later)	AV block, recent decompensation, dialysis	Permanent AF, NYHA IV or unstable HF, bradycardia, AV block	Unsuitable for flecainide
AF pattern	Persistent AF	50% persistent	Persistent AF	69% AF episode longer than 2 days	Persistent AF	Persistent AF	2/3 persistent	Not available but 25% were in AF at time of randomization	Persistent AF
Duration of AF at baseline (years)	0.3 (0.3)	<0.5	0.9	35% first episode of AF	0.5 (0.2)	74% < 1	<1	Not available	2.3
Rhythm control intervention	Amiodarone	Amiodarone	Antiarrhythmic drugs	Antiarrhythmic drugs	Antiarrhythmic drugs	Sotalol, amiodarone	Amiodarone	Dronedarone	Flecainide (short and long term)
Comparator therapy	Rate control (diltiazem)	Sotalol or propafenone	Rate control	Rate control	Rate control	Placebo	Rate control	Placebo	No antiarrhythmic drug
Primary endpoint	Recurrent AF	Recurrent AF	Cardiovascular death, HF, stroke, bleeding, pacemaker, or SAE	Death	MACCE	Recurrent AF	Cardiovascular death	Cardiovascular hospitalization or death	Recurrent AF
Method for detecting recurrent AF	24-h Holter every 3 months	Regular ECG during follow-up	Regular ECG during follow-up	Not specified	Regular ECG upon follow-up	Monthly ECG	Yearly ECG	Yearly ECG	Daily telemetric ECG
Sinus rhythm maintenance	56% at 52 weeks on amiodarone, 10% on diltiazem	40% at 2 years on sotalol/prop, 60% on amiodarone	38% in rhythm control group, 10% in rate control during 2.3 years follow-up	60% in active group, 30% in control group at 5 years	40% at 12 months, 26% at 24 months in active group	At 12 months: 52% amio, 32% sotalol, 13% placebo	At 48 month visit: 70% (amio) vs. 30% (control), 58% of rhythm control group had AF during follow-up	Median time to first AF recurrence 737 days in dronedarone group and 498 in placebo	60% (flecainide) vs. 40% (control) at 6 months
Outcomes	Improved 6MWT in rhythm control patients	No difference in QoL between groups	No difference in mortality or QoL between groups	No difference in mortality or QoL between groups	No difference in MACCE. Reduced recurrent AF	No difference in mortality or QoL between groups	No difference in mortality or QoL between groups	Lower mortality and less hospitalizations in patients randomized to dronedarone	Improved quality of life in all groups

All studies found reduced AF recurrences in patients randomized to rhythm control therapy. Several studies reported improved quality of life in patients with successful sinus rhythm maintenance, e.g. in SAFE-T and AF-CHF. AAD antiarrhythmic drug. 6MWT, six minute walking test; QoL, quality of life.

## Effectiveness and safety of rhythm control therapy

The success of rhythm control therapy depends on multiple factors including the number, type, and severity of underlying conditions, age, gender, adherence to antiarrhythmic drug therapy, and factors related to the quality of the AF ablation procedure.^2^^,^^8^^,^^20^ Furthermore, AF recurrence rates depend on the intensity of electrocardiogram (ECG) monitoring and duration of follow-up.^15^ Thus, comparing absolute recurrence rates between studies and comparisons to historical controls can be misleading (*Table 1*).

## Effectiveness and safety of antiarrhythmic drug therapy

On average, antiarrhythmic drugs double the proportion of patients who maintain sinus rhythm. Amiodarone is more effective than other antiarrhythmic drugs in maintaining sinus rhythm, and catheter ablation is more effective than antiarrhythmic drugs.^8^ The long-term complication rates of antiarrhythmic drug therapy are comparable to complications in patients treated with AF ablation.^9^^,^^21^ Although amiodarone has been associated with adverse outcomes in non-randomized analyses of patients at very high risk,^22^ the safety of antiarrhythmic drug therapy found in recent randomized trials in patients with AF attenuates historical safety concerns,^9^^,^^21^ particularly in patients with heart failure.^23^ Unlike earlier trials of antiarrhythmic drugs compared to placebo or rate control therapy (*Table 1*),^23–25^ antiarrhythmic drug therapy with dronedarone was associated with reduced cardiovascular hospitalizations and cardiovascular deaths compared to placebo.^26^ The same substance, dronedarone, used as a rate-controlling agent, was associated with higher rates of heart failure, stroke, and cardiovascular death in patients with permanent AF in the PALLAS trial.^27^ Patients included in PALLAS were not considered suitable for rhythm control therapy, did not receive interventions to restore sinus rhythm (e.g. cardioversion, AF ablation) and had severe heart failure. Hence, they were deprived of any potential to benefit of sinus rhythm. Patients treated with dronedarone in ATHENA, in contrast, received that therapy to restore sinus rhythm. Taken together, these data may suggest that the beneficial effects found in ATHENA could be associated with its rhythm controlling effect, but more data are needed.

Antiarrhythmic drugs are also effective after AF ablation. Two recent randomized studies (AMIO-CAT^28^ and POWDER-AF^29^) showed that adding antiarrhythmic drug therapy to AF ablation improves sinus rhythm maintenance for the duration of therapy. This synergistic effect of antiarrhythmic drugs with AF ablation reflects the common (approximately 50% of patients) use of antiarrhythmic drugs 1 year after AF ablation.^30^ A substudy within AMIO-CAT measuring brain natriuretic peptide suggested that biomarkers may improve identification of patients at risk for recurrent AF,^31^ pointing potentially towards personalized or stratified selection of patients for specific rhythm control therapies.^32^

## Effectiveness and safety of atrial fibrillation ablation

Initially evaluated in young patients with highly symptomatic AF (mean age around 55 years) who were refractory to antiarrhythmic drug therapy, AF ablation maintains sinus rhythm better than antiarrhythmic drugs.^33^^,^^34^ This was confirmed in CABANA.^9^ A meta-analysis of randomized trials (6167 patients) found that AF ablation achieves freedom from recurrent AF in approximately half of the patients [53% (46–60), mean (95% confidence interval, CI)], with slightly higher recurrence rates in patients with chronic forms of AF.^35^ Periprocedural complications occur in ca. 5% of patients (7.8% in EORP AF ablation, 4.8% in CABANA), including tamponade (ca. 1%), stroke, or transient ischaemic attack (ca. 0.5–1% in anticoagulated patients), access site complications (ca. 2–3%), and death (<1%).^30^^,^^36–38^ Reablation is performed in 20–50% of patients undergoing a first AF ablation. During long-term follow-up for up to 10 years, up to 60% of AF ablation patients remain free of clinically relevant recurrences of AF (with around three-fourths in sinus rhythm after 1 year), and approximately half of these patients receive combination therapy with antiarrhythmic drugs.^39–41^ Whether additional ablation strategies improve these outcomes needs to be investigated.^42^

## Atrial fibrillation ablation compared to antiarrhythmic drug therapy after CABANA

CABANA was designed to test whether AF ablation can reduce mortality compared to antiarrhythmic drugs in patients with AF in need for rhythm control therapy and with stroke risk factors.^43^ In early 2013, a planned, blind data review identified slow enrolment and lower event rates than anticipated. This resulted in a change in primary endpoint from all-cause mortality to a composite of death, disabling stroke, serious bleeding, or cardiac arrest. In addition, the sample size was reduced. The results have just been reported^9^: Of the 2204 patients randomized (median age, 68 years; 37% female; 57% persistent AF), 89.3% completed the trial. In patients randomized to AF ablation, 91% underwent the procedure, while AF ablation was performed in 27.5% of the patients randomized to drug therapy, in line with expectations at the start of the trial.^43^ Safety of rhythm control therapy was good in this elderly patient population (mean age 68 years), with low complication rates in both arms: Patients randomized to AF ablation experienced tamponade (0.8%), haematomas (2.3%), and pseudoaneurysms (1.1%). Patients randomized to antiarrhythmic drug therapy experienced thyroid disorders (1.6%) and proarrhythmia (0.8%). The primary outcome was not different between groups.^9^ Over a median follow-up of 48.5 months, the primary endpoint occurred in 8.0% of patients randomized to AF ablation, and in 9.2% of patients randomized to antiarrhythmic drug therapy [hazard ratio (HR) 0.86, 95% CI 0.65–1.15; *P* = 0.30]. Key secondary outcomes were not different between random groups, including all-cause mortality was 5.2% and 6.1% (HR 0.85, 95% CI 0.60–1.21; *P* = 0.38), death or cardiovascular hospitalization rates were 51.7% and 58.1% for (HR 0.83, 95% CI 0.74–0.93; *P* = 0.001). Recurrent AF was less common in patients randomized to AF ablation in the subgroup of 1240 patients undergoing systematic ECG monitoring (HR 0.52, 95% CI 0.45–0.60; *P* < 0.001). Both treatment groups showed improved quality of life, as assessed by the Atrial Fibrillation Effect on Quality of Life (AFEQT) summary score and the Mayo AF-Specific Symptom Inventory (MAFSI). Patients randomized to catheter ablation showed a greater improvement in quality of life (mean difference of 5.3 points).^44^ This greater effect of AF ablation on quality of life is consistent with the main finding of the Swedish CAPTAF trial.^45^

Similar to other observational data sets, on-treatment analysis suggested improved outcomes in patients undergoing AF ablation. These findings are additionally supported by a recent study using a large US administrative database of routine patient data, analysing patients who meet the CABANA inclusion criteria.^46^ Unknown and known confounders, censoring of events—either intentionally by study design or unintentionally because of loss to follow-up—, self-selection of low risk patients to cross over to ablation, and immortal time bias are some of the sources of bias that can explain these findings.^47^

## Rhythm control therapy in patients with atrial fibrillation and heart failure

Atrial fibrillation and heart failure (AF+HF) frequently coexist and this is associated with high morbidity and mortality.^48^ To improve outcomes, restoring and maintaining sinus rhythm has been proposed in patients with AF+HF. Amiodarone is the only antiarrhythmic drug with sufficient safety data in patients with reduced left ventricular ejection fraction.^8^ Large randomized trials of antiarrhythmic drugs compared to rate control in patients with AF+HF did not find differences in all-cause mortality, cardiovascular mortality, or heart failure hospitalizations.^23^^,^^49^^,^^50^ Likewise, patients who maintain sinus rhythm (‘successful rhythm control therapy’) did not have better survival than those with recurrent AF.^50^ Several small case series and controlled trials found that patients undergoing AF ablation have improved left ventricular function, often using echocardiography to assess left ventricular (LV) function (*Table 2*): four out of five relatively small studies found improved left ventricular function in patients with AF+HF randomized to AF ablation (*Table 2*),^51–59^ largely seen in trials that assessed left ventricular function by echocardiography, which is less reliable in AF than in sinus rhythm.^60^ There were associated improvements in exercise capacity and brain natriuretic peptide (BNP) levels (*Take home figure*, bottom panel). Improved exercise capacity and to some extent improved left ventricular function, but not lower BNP, could be partially explained by bias in unblinded trials. These effects have been extrapolated with a certain enthusiasm.^61^ The largest trial comparing AF ablation with ‘medical therapy’ (mostly rate control, but including antiarrhythmic drugs) in patients with AF+HF is CASTLE-AF (*Table 2*).^10^ The quality of rate control therapy may have affected changes in LV function in the control group of the published trials that used rate control as comparator. Thirty-four of the 363 randomized patients were lost to follow-up despite an implanted device allowing home monitoring. In the remaining patients, catheter ablation reduced mortality and HF hospitalizations (28.5% compared with 45%), but had no effect on all-cause hospitalizations and stroke. Details of the drug therapy given to patients randomized to ‘medical therapy’ have not been published. One-third of the patients assigned to medical therapy were on antiarrhythmic drugs at their final follow-up, 22% were in sinus rhythm at 60 months (compared to 63% in the AF ablation arm, *Table 2*). In line with these findings, the recent update of the AHA/ACC/HRS guidelines for AF included a Class IIb recommendation for AF ablation in patients with heart failure. So far, there is no information about outcomes following catheter ablation for AF in patients with heart failure and a preserved ejection fraction. Despite these limitations, CASTLE-AF and the AATAC trial^62^ contribute evidence that selected patients with AF+HF benefit from AF ablation (*Table 2*),^51–59^ but open questions remain regarding selection of adequate patients and validity of the findings in ‘all-comer’ patients. More research is needed to determine the effect of AF ablation on cardiovascular outcomes in patients with AF+HF.

**Take home figure:**
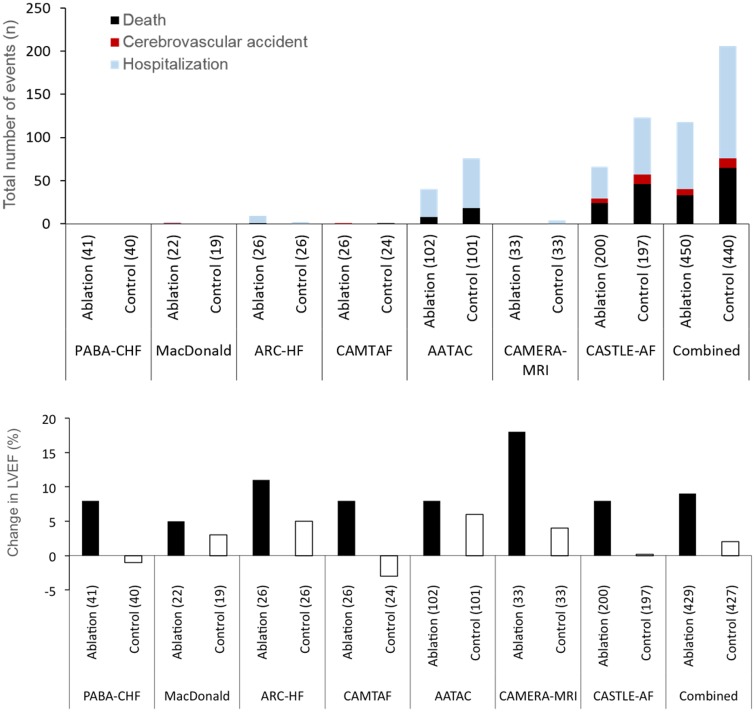
AF ablation may affect cardiovascular outcomes (top panel) and appears to improve left ventricular function (bottom panel) in selected patients with Atrial Fibrillation and Heart Failure. Further evidence is needed to underpin these hypothesis-generating findings.

**Table 2:** Randomized studies comparing pharmacological rate or rhythm control, or, in PABA-CHF, AV nodal ablation and biventricular pacing, with catheter ablation in patients with AF and systolic dysfunction with reduced ejection fraction

	PABA-CHF	MacDonald	ARC-AF	CAMTAF	AATAC	CAMERA-MRI	CASTLE-AF
Year of publication	2008	2011	2013	2014	2016	2017	2018
Number of patients	81	41	52	50	203	66	363^a^
Age	61	63	63	58	61	61	64
Sex	>80% male	78% male	>80% male	96% male	74% male	91% male	86% male
Type of patients	NYHA II–III, LVEF <40%	NYHA II–IV, LVEF <35%	NYHA II–IV, LVEF <35%	NYHA II–IV, LVEF <50%	NYHA II–III, LVEF <40%, dual-chamber ICD or CRT	NYHA II–IV, LVEF <45%^b^	NYHA II–IV, LVEF <35%, dual-chamber ICD or CRT
Exclusion criteria	Post-operative AF, reversible causes of AF or HF, prior AF ablation	Paroxysmal AF, QRS duration >150 ms, myocarditis	Reversible causes of AF and HF	Previous AF ablation, reversible HF cause	Amiodraone therapy, AF <3 months duration, reversible AF	Paroxysmal AF, contraindications to ablation or MRI, ischaemic cardiomyopathy	Prior AF ablation, LA diameter >60 mm
Proportion with ischaemic HF aetiology	70%	49%	33%	26%	64%	0%	46%
AF pattern	52% paroxysmal	100% chronic	100% chronic	100% chronic	100% chronic	100% chronic	33% paroxysmal
Duration of AF at baseline	48 months	44 months	51 months	24 months	9 months	22 months	Not known
Comparator therapy	Rate control (AV nodal ablation + biventricular ICD)	Pharmacological rate control	Pharmacological rate control	Pharmacological rate control	Rhythm control with amiodarone	Pharmacological rate control	Mixture of rate control and rhythm control
Primary endpoint	Composite of LVEF, 6MWT distance, and MLHFQ score	Change in LVEF from randomization to last study visit	Peak VO_2_	LVEF at 6 months	Freedom from AF, AFL, or AT of >30 s duration off AAD at follow-up	Change in LVEF from baseline at 6 months on cardiac MRI	Composite of all-cause mortality or worsening of HF requiring unplanned hospitalization
Method for AF recurrence assessment	External loop recorder (AF ablation patients only)	24-h Holter at baseline, 3 and 6 months	48-h Holter at 6 and 12 months	48-h Holter at 1, 3, and 6 months (and 12 months in AF ablation patients)	Device interrogation at 3, 6 12, and 24 months	Implanted loop recorder in AF ablation patients	Device interrogation at 3, 6, 12, 24, 36, 48, and 60 months
Sinus rhythm maintenance at end of follow-up	88%	50%	88%	73%	70%	75% (56% without antiarrhythmic drugs)	63%
Outcomes	Improved LVEF, 6MWT distance and QoL (MLHFQ) in AF ablation patients	No difference in LV or RV function (measured by cardiac MRI), 6MWT, or BNP between groups	Improved exercise performance, QoL and BNP levels in AF ablation patients	Greater improvement in LVEF, better exercise performance, lower BNP, and improved QoL AF ablation patients	Less unplanned hospitalization, lower mortality, greater improvement of LVEF, 6MWT distance, and QoL (MLHFQ) in AF ablation patients	Greater improvement of LVEF at 6 months in AF ablation patients	Less mortality and HF hospitalizations in AF ablation patients

a Number of randomized patients.

b 6-Min walk distance and serum brain natriuretic peptide did not support the presence of heart failure in all patients. 6MWT, six minute walking test; AF, atrial fibrillation; BNP, brain natriuretic peptide; CRT, cardiac resynchronization therapy device; ICD, implantable defibrillator; LA, left atrium; LV, left ventricle; LVEF, left ventricular ejection fraction; MLHFQ, Minnesota Living with Heart Failure Questionnaire; MRI, magnetic resonance imaging; NYHA class, New York Heart Association functional class; QOL, quality of life; RV, right ventricle.

## Rhythm control therapy and stroke

The clear association of AF and ischaemic stroke may suggest that maintaining sinus rhythm can help to prevent strokes. There is no signal for reduced strokes in the earlier ‘rate vs. rhythm’ studies (*Table 1*), including the reasonably large AF-CHF trial.^23^ There were only three and seven disabling strokes in each arm in CABANA, without differences between groups.^9^ Interestingly, in a *post hoc* analysis of the ATHENA trial (*Table 1*), patients randomized to dronedarone had a lower risk of stroke or transient ischaemic attack (1.2% vs. 1.8%).^26^ A retrospective, propensity-score matched analysis of a subset of AF patients taken from the Swedish patient registry also suggested that AF ablation may be associated with a lower incidence of ischaemic stroke.^63^ This is similar to propensity-matched patient comparisons in the largest health maintenance organization in Israel, comparing 969 AF patients undergoing AF ablation to 3772 AF controls.^64^ These analyses are prone to several biases, including known, unmeasured and unknown confounders, and others.^63^

## Rhythm control therapy and cognitive decline

Atrial fibrillation is associated with cognitive dysfunction and dementia. Anticoagulation appears to reduce dementia in patients with AF in a nationwide cohort analysis.^65^ While it is unlikely that antiarrhythmic drug therapy causes cerebral complications (stroke, transient ischaemic attack, or cognitive decline), there is a peri-procedural risk of ischaemic stroke (0.3–1%) as well as a risk of magnetic resonance imaging (MRI)-detected clinically silent ischaemic brain lesions in patients undergoing AF ablation.^37^ This can increase brain damage and subsequently lead to cognitive decline.^37^ Interestingly, the AXAFA–AFNET 5 study found small MRI-detected brain lesions in ca. 30% of patients undergoing a first AF ablation on continuous anticoagulation, but also detected an improved cognitive function as assessed by Montreal Cognitive Assessment (MoCA) 3 months after AF ablation.^38^

Rhythm control therapy may reduce AF-related stroke risk by reducing AF burden and subsequent improvement in atrial cardiomyopathy,^66^ potentially reducing silent embolic lesions, and possibly improving perfusion and metabolism of the brain. A large retrospective observational study found a lower rate of new-onset dementia in 4212 patients undergoing AF ablation compared to 16 848 non-ablated AF patients, while a substudy in the randomized AFFIRM trial did not find a difference in cognitive function between patients randomized to rate or rhythm control therapy, while the AXAFA study found improved cognitive function in 674 patients 3 months after AF ablation compared to baseline.^38^ The possible cognitive benefits of restoring sinus rhythm in AF patients can be attenuated by atrial cardiomyopathy and by concomitant cardiovascular conditions and other unknown confounders that can cause brain damage, stroke, and cognitive dysfunction in the absence of AF.^66^^,^^67^ Unfortunately, neither CABANA nor CASTLE-AF reported cognitive function outcomes. Ongoing research such as the case–control DIAL-F cohort (NCT01816308) and the randomized EAST-AFNET 4 trial^68^ will provide further information on the impact of rhythm control therapy including AF ablation on cognitive function.

## Rhythm control therapy and atrial cardiomyopathy

The term ‘atrial cardiomyopathy’ summarizes the structural, architectural, contractile, or electrophysiological changes in diseased atria.^66^ Cardiovascular diseases (e.g. hypertension, heart failure, valvular heart disease, ischaemic heart disease, or diabetes) but also ageing can contribute to an atrial cardiomyopathy. Atrial fibrillation itself accelerates the underlying disease processes, thus contributing to atrial cardiomyopathy.^69^ Left atrial enlargement, a summative clinical proxy for atrial cardiomyopathy, is partially reversed after AF ablation.^7^^,^^70^^,^^71^ Early rhythm control therapy, including AF ablation, has been suggested to slow these processes, thereby simplifying rhythm control therapy and potentially improving long-term outcomes.^68^ Hence, early rhythm control therapy could slow atrial cardiomyopathy. However, this hypothesis requires confirmation in further studies and trials.

## Summary and conclusions

Recent randomized trials and observational data sets including CASTLE-AF and CABANA provide important reassurance on the safety of rhythm control therapy in contemporary patients with AF, including in elderly patients with concomitant cardiovascular diseases. The data confirm the superior effectiveness of AF ablation compared to antiarrhythmic drugs to restore and maintain sinus rhythm, and demonstrate that antiarrhythmic drugs remain effective after AF ablation. Several smaller studies suggest that AF ablation can improve left ventricular function assessed by echocardiography in selected patients with AF and heart failure. Further studies to investigate the impact of rhythm control therapy on LV function in different, clearly defined subsets of patients with AF are warranted. The effects of rhythm control therapy on cardiovascular death, stroke, heart failure, acute coronary syndromes, as well as secondary outcomes such as left atrial, ventricular, and cognitive function require further research, such as the on-going EAST–AFNET 4 trial.^68^
